# When a vehicle becomes a weapon: intentional vehicular assaults in Israel

**DOI:** 10.1186/s13049-016-0338-9

**Published:** 2016-12-28

**Authors:** Gidon Almogy, Asaf Kedar, Miklosh Bala

**Affiliations:** Department of Surgery and Trauma Unit, Hadassah-Hebrew University Medical Center, Jerusalem, Israel

**Keywords:** Intentional vehicular trauma, Pedestrian trauma, Terrorism, Multiple casualty incidents, Head trauma

## Abstract

**Background:**

We have recently witnessed an epidemic of intentional vehicular assaults (IVA) aimed at pedestrians. We hypothesized that IVA are associated with a specific injury pattern and severity.

**Methods:**

Retrospective analysis of prospectively acquired data of patients injured following IVA from October 2008 to May 2016 who were admitted to the Hadassah Level I trauma center in Jerusalem, Israel. Comparison of injury parameters and outcome caused by vehicular attacks to non-intentional pedestrian trauma (PT). Measured outcomes included ISS, AIS, injury pattern, ICU and blood requirements, participating teams, length of stay, and mortality.

**Results:**

There were 26 patients in the IVA group. Mean age in the IVA group was significantly younger and there were more males compared to the PT group (24.7 ± 13.3 years vs. 48.3 ± 21.3, and 81% vs. 52%, respectively, *p* < 0.01). Lower extremity (77% of patients), followed by head (58%) and facial (54%) injuries were most commonly injured in the IVA group, and this was significantly different from the pattern of injury in the PT group (54, 35, and 28%, respectively, *p* < 0.05). Mean ISS and median head AIS were significantly higher in the IVA group compared with the PT group (23.2 ± 12.8 vs. 15.4 ± 13.8, *p* = 0.012, and 4.5 vs. 3, *p* = 0.003, respectively). ICU admission and blood requirement were significantly higher in the IVA group (69% vs. 38%, and 50% vs. 19%, *p* < 0.01). Mortality was significantly higher in the IVA group (4 patients, 15%, vs. 3 patients, 4%, respectively, *p* = 0.036) and was caused by severe head trauma in all cases.

**Discussion:**

The severity of injury and mortality rate following IVA are higher compared with pedestrian injury. The pattern of injury following IVA is significantly different from non-intentional pedestrian trauma.

**Conclusions:**

IVA results in higher mortality than conventional pedestrian trauma secondary to more severe head injury. More hospital resources are required following IVA than following conventional road traffic accidents.

## Background

Since the first recorded pedestrian fatality in 1896 in Europe and in 1899 in the Americas, there were countless accidents causing injuries and fatalities from motor vehicles. In the era of modern motor vehicles, we have acquired knowledge of the mechanism of injury and pathophysiology of injury, developed treatments protocols and prevention. These accidents were almost universally unintentional. Injury caused by a motor vehicle driven intentionally into a pedestrian crowd with an intention to cause harm has recently become an uncommon and novel method of terrorism.

**Fig. 1:**
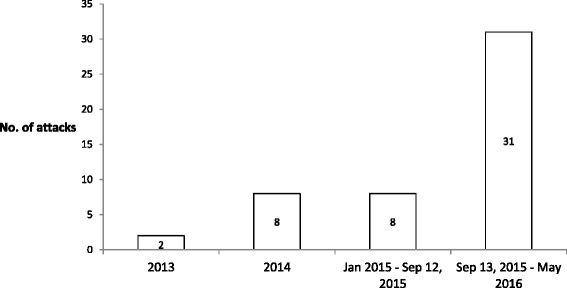
The number of intentional vehicular assaults over recent years, including those with and without casualties. September 13, 2015 marks the beginning of the ‘Lone Wolf Intifada’

This method of attack was first seen in Israel in 1987 during the first “Intifada” when a car was intentionally driven to into a group of soldiers inflicting severe injury. Over the past decades, there have been random attacks. Since September 2015, we have witnessed a surge of vehicular assaults (Fig. 1). We suggest the term “intentional vehicular assault” (IVA) to describe this specific type of violence. IVA is a novel method used by the lone attacker. Radicals who embark on individual terrorist missions with little or no logistical support characterize this ‘lone wolf’ phenomenon [1].

Terror acts in the Middle East have evolved from stabbings and firearm attacks to suicide bombings. Multi-dimensional injury caused by terrorist bombings has been described in the literature [2–4]. Several manuscripts describe the experience acquired in Israel and analyze the physical factors that are responsible for injury following an explosion, the resulting injuries and the appropriate medical care [5–7].

**Fig. 2:**
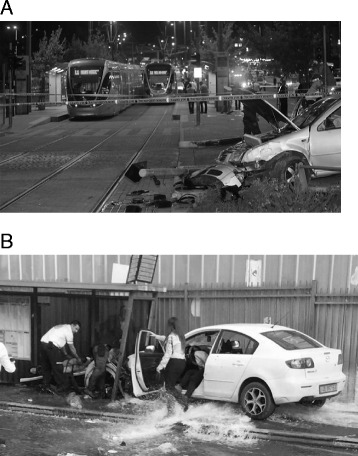
Typical pictures from the scenes of two IVA’s in Jerusalem, on a tram station **a** and bus stop **b**. Note signs of a high-energy attack such as a knocked down electrical pole (**a**), run over fire hydrant, and damage to bus stop (**b**)

From February 2008 to May 2016 twenty-nine IVA with casualties took place in Israel. Civilians were targeted in 15 attacks (52%), and security personnel including police officers, border police and soldiers in 14 attacks (48%). Vehicles used to carry out these attacks included private vehicles (n=18, 62%), heavy mechanical vehicles (n=5, 17%), commercial vehicles (n=4, 14%), and trucks (n=2, 7%). Some of the popular sites included main city streets (n=12, 41%), road blocks (n=7, 24%), and tram and bus stations (n=6, 21%) (Fig. 2).

Our primary goal was to describe and characterize this evolving type of violence and the pattern of injury that it causes. Our secondary goal was to compare injury severity and outcome following IVA with civilian, non-terror related pedestrian trauma.

## Methods

Data regarding vehicular attacks in Israel was collected from official governmental sites, published information and media reports.

Data regarding admitted patients was retrieved from the Hadassah Hospital Trauma Registry. We retrospectively analyzed prospectively collected data of all victims of vehicular attacks who were admitted to the Ein Kerem Campus, Hadassah Hospital level I Trauma Center, in Jerusalem, Israel, from October 2008 to May 2016. The registry data include all casualties admitted to the emergency department (ED) and hospitalized, succumbed in the ED, or transferred to another hospital following injury. The registry does not include casualties who died at the scene or on way to the hospital, patients who were not admitted, or patients who were admitted only 72 h or later following the event.

Only patients hospitalised following IVA were included into the study group. The information consisted of the number hospital admissions, ISS, region injured, surgical interventions, ICU admission, ICU and hospital length of stay, and mortality. All injuries were divided into common anatomic regions (head, face, chest, abdomen, pelvis, upper and lower extremities, spine and burns). Abbreviated injury score (AIS) was calculated according to injured organs.

The data of the IVA group was compared to 81 patients between the ages of 18 and 60 who were injured as pedestrians in a civilian, non-trauma related setting during 2014 (ICD-10, Pedestrian injured in collision with car, pick-up truck or van; V03). These 81 patients were defined as the pedestrian trauma (PT) group. The study was approved by Hadassah IRB and consent waiver was received.

There are 3 hospitals in Jerusalem serving a population of over 800,000 inhabitants. The Ein Kerem Campus of the Hadassah Hospital is a level I Trauma Center, Shaarei-Zedek is level II and the Mount Scopus Campus (Hadassah Hospital) is level III. EMS crews are instructed to evenly distribute severely injured trauma patients among the level I and II hospitals. Civilian and military casualties with moderate and severe head injuries are preferentially directed to the Ein Kerem Campus.

### Statistical analysis

Data are presented as mean and standard deviation (SD) or number of patients and percentage. The chi-squared test was used to compare proportions and the Student’s *t*-test was used to compare continuous non-parametric variables. A *p* value of 0.05 or less was considered statistically significant. Statistical analysis was performed using SPSS version 11.5 (Statistical Package for Social Science, Chicago, Ill).

## Results

Our level I trauma center received 26 victims of 13 IVA. The median number of patients admitted to our trauma center following an attack was one, with a range of 1 to 5 victims per attack. Their data are shown in Tables 1 and 2.

**Table 1:** Demographic and admission characteristics in both types of trauma

	Vehicular assaults (*n* = 26)	Pedestrian trauma (*n* = 81)	*P* value
Age, mean^a^	24.7 ± 13.3	48.3 ± 21.3	<0.001
Sex (males)	21 (81)	42 (52)	0.009
ICU admission	18 (69)	31 (38)	0.006
ICU LOS, mean (days)^a^	4.3 ± 6.8	7.8 ± 9	NS
LOS, mean (days)^a^	11.9 ± 13.5	11.5 ± 12.7	NS
Required surgery	17 (65.4)	38 (46.9)	0.1
Number of surgical procedures per patient, mean^a^	1.7 ± 0.7	1.2 ± 0.5	0.007
Received blood in first 24 h	13 (50)	15 (19)	0.0015

The Student’s *t*-test and chi-squared test were used for statistical analysis as appropriate

*Abbreviations*: *LOS* length of stay, *ICU* intensive care unit

^a^Data shown as number (and percentage) and mean ± standard deviation

**Table 2:** Regions of body which were injured in both types of trauma

	Vehicular assaults (*n* = 26)	Pedestrian trauma (*n* = 81)	*p* value
ISS, mean^a^	23.2 ± 12.8	15.4 ± 13.8	0.012
Head	15 (58)	28 (35)	0.036
Face	14 (54)	23 (28)	0.0176
Chest	3 (12)	25 (31)	0.051
Abdomen	3 (11)	15 (19)	NS
Lower extremities	20 (77)	44 (54)	0.04
Upper extremities	8 (31)	29 (36)	NS
Spine	7 (27)	9 (11)	0.049
Skin	7 (27)	11 (14)	NS
Pelvis	3 (12)	26 (32)	0.04
Vascular	3 (12)	0 (0)	0.0019
Ocular	2 (8)	4 (5)	NS

The Student’s *t*-test and chi-squared test were used for statistical analysis as appropriate

*Abbreviations*: *ISS* injury severity score

^a^Data shown as number (and percentage), and mean ± standard deviation

### IVA vs. PT

Casualties in the IVA group were predominantly male and significantly younger than patients in the PT group (Table 1). Seven patients in the IVA group arrived at the ED intubated (27%), significantly more than in PT group (*n* = 6, 7%, *p* = 0.008). The number of patients who required surgery was not different between the groups. However, the mean number of surgical procedures per patient was significantly higher in the IVA group (1.65 ± 0.7 vs. 1.2 ± 0.5, *p* = 0.007). Significantly more patients in the IVA received blood (Table 1), but the number of blood products per patient was not different.

Head, face, spinal and lower extremity injuries were significantly more common in the IVA group (Table 2). Median head AIS was significantly higher in the IVA group (4.5 vs. 3, *p* = 0.003, respectively). Following IVA the teams participating included orthopedics (*n* = 12), neuro-surgical (*n* = 5), spine surgery (*n* = 1) and plastics surgery (*n* = 1). Following PT the teams participating included orthopedics (*n* = 29), neuro-surgical (*n* = 6), general surgery (*n* = 6), plastics surgery (*n* = 2) and ear nose and throat (*n* = 2). Combined head, lower extremity and pelvic fractures were noted only in 2 patients in IVA group (8%) and 4 patients (5%) in the PT group (*p* = NS).

### Mortality

Mortality was significantly higher in the IVA group compared with the PT group (4 of 26 patients [15%], vs. 3 of 81 patients [4%], *p* = 0.036). In the IVA group all four deaths were caused by severe head trauma on days 1 (*n* = 1), 2 (*n* = 1), and 4 (*n* = 2) of admission. The causes of death for the three patients in the PT were severe head trauma (day 1), and multi-organ failure (days 28 and 32).

## Discussion

IVA have recently emerged as the preferred method of attack by unarmed and untrained, mostly religiously motivated individuals, also termed ‘lone wolves’ [1]. To date, these attacks have occurred mostly in the Middle East and Europe. Since very little training and preparation is necessary to carry out these attacks, we can expect more similar attacks on a worldwide basis. An internet search showed that in Israel the majority of assaults involved civilian cars although some of them used heavy construction equipment, making the attacks more lethal. The number of casualties per attack varied widely and ranged from one to 70, with a median of 3 causalities per attack. Seven attacks (24%) resulted in deaths, with a range of 1 to 3 deaths per attack. Two attacks involved drivers ramming their vehicles into pedestrians on the main street, abandoning the vehicle, and continuing their assault by stabbing passers-by. The city of Jerusalem was the most common target for IVA. Fourteen attacks (48%) occurred within the boundaries of the city and seven additional attacks (24%) took place in its immediate vicinity (within a range 20 km).

The cardinal finding of this study is that the pattern and severity of injury following IVA is significantly different from non-intentional pedestrian injury. Our data show that ISS is significantly higher following IVA. This higher ISS is due to more severe head injury that is characterized by higher head AIS. The higher frequency of lower extremity injuries during IVA compared to PT further supports our assumption that IVA causes a different pattern of injury.

Age and severity of head injury exert the strongest impact on prognosis and mortality following motor vehicle accidents [8]. Strikingly, the IVA group had significantly higher mortality compared to civilian pedestrians (15% vs. 4%, *p* = 0.036), and this difference was secondary to head trauma. All 4 deaths in the IVA group were caused by severe head injuries, compared to only one death in the PT group. This correlates well with the fact, that it is exactly these two factors, mortality and head trauma, in which IVA and the control group differ the most in our analysis.

In addition to a significantly higher rate of severe head injury, the IVA group also required significantly more pre-hospital interventions. The intubation rate was almost four-fold higher in the IVA group (27% vs. 7%). The severity of injuries, specifically head injury, and despite the scoop and run regime of emergency teams, and short transport times, could be an explanation for the higher intubation rate. Similar findings are apparent concerning in-hospital surgical procedures. The IVA group presented severe injuries making surgery necessary in nearly two-thirds of cases. This is in contrast to pedestrians who underwent operations in less than half of cases.

Our data show that victims of IVA were younger. This represents the younger age of security personnel, compared to the average age of civilian pedestrians. A similar age pattern was observed in previous studies on suicide bombing attacks published from our institution [9, 10].

As a consequence of a non-intentional related car accident, the pedestrian is hit by a car which is decelerating, and the victim is often shoved away with injury to the extremities and pelvis, but with less severe head trauma. At this point of time, we can only try to understand the mechanism injury following an IVA. By studying available sources such as video clips and witness reports, we anticipate that deliberate acceleration of the vehicle into an upright pedestrian may lead to high energy trauma to the lower extremities and to severe head trauma when and the victim is hurled towards the vehicle. In fact, our results showed this particular type of injury pattern in the IVA group (lower extremities and head trauma), as opposed to more pelvic injuries in the PT group. More studies are necessary to clarify this phenomenon.

This general pedestrian injury pattern is supported by the classic theory of pedestrian versus motor vehicle kinematics [11]. However, more recent investigations demonstrated significant inter-individual variations in common pedestrian injury combinations that are influenced by several factors (e.g., vehicle type, body region of first impact, main impact direction etc.) [12–15]. Our data confirms this conclusion. We were unable to identify the classic pedestrians “fatal triad” of injuries as described by Farley and Waddell for severely injured victims [16, 17]. Only a few patients had all three injuries, and there were no differences between the groups.

Previous reports have shown that terrorist attacks strain and challenge hospital resources when compared to civilian trauma. Our data show that the number of surgical procedures per patient, ICU and blood transfusion requirements, were significantly higher following IVA compared with PT. The severity of head injury among patients with head injuries was also significantly higher following IVA. Thus, more resources are needed to manage victims of intentional trauma compared to non-intentional trauma.

The median number of casualties who were admitted to the ED following an IVA was relatively small (range of 1–5 victims/attack) when compared with the number of casualties following other types of terror acts such as suicide bombing attacks. However, four IVA resulted in multiple casualties and can be defined as multiple casualty incidents (MCI, defined as ≥10 casualties arriving at hospital). In fact, there were 70 casualties when a bulldozer rammed into a crowd on a Jerusalem main street. Likewise, there were more than 200 casualties following the Nice (France) attack (July 14, 2016) which involved a truck. Admitting hospitals are required to prepare for an MCI following IVA.

## Conclusions

In conclusion, victims of IVA are characterized by severe head injuries and a higher subsequent mortality when compared to pedestrians involved in non-intentional road traffic collisions. Increased hospital resources are required to appropriately manage IVA. Attention should be given to constructing anti-vehicle barriers and other passive protective means to shield pedestrians in popular sites which may serve as potential terrorist targets.

## Abbreviations

AIS: Abbreviated injury score
ED: Emergency department
ICU: Intensive care unit
ISS: Injury severity score
IVA: Intentional vehicular assault
LOS: Length of stay
MCI: Multiple casualty incidents
PT: Pedestrian trauma
